# Is Subdural Peritoneal Shunt Placement an Effective Tool for the Management of Recurrent/Chronic Subdural Hematoma?

**DOI:** 10.7759/cureus.613

**Published:** 2016-05-18

**Authors:** Andres M Alvarez-Pinzon, Alan A Stein, Jose E Valerio, Victor Delgado, Jennifer A Escalante, Nithia Lopez, Aizik L Wolf

**Affiliations:** 1 Miami Neuroscience Center, Larkin Community Hospital; 2 Biotechnology - NeuroOncology, Biotechnology Advanced Academic Programs | Johns Hopkins University; 3 Charles E. Schmidt College of Medicine, Florida Atlantic University; 4 Neurosurgery, Florida International University

**Keywords:** subdural hematoma, shunt, craniotomy, mental status, trauma

## Abstract

**Objective:**

To describe a surgical technique and to report using a retrospective study the efficacy of peritoneal shunts for the treatment of recurrent/chronic subdural hematoma (CSDH). We describe the considerations, complications, and outcomes related to this technique.

**Methods:**

In a retrospective cohort study, 125 charts with a diagnosis of subacute/chronic subdural hematoma were assigned for evaluation. Of the charts reviewed, 18 charts were found from subjects with a diagnosis of recurrent sub-acute or chronic subdural hematoma. All patients had undergone initial surgical treatment of their condition followed by peritoneal shunt placement to help alleviate intracranial pressure. Factors including the age, size of subdural hematoma, number of previous events, BMI, complications, survival, and clinical course were analyzed.

**Results:**

After subdural peritoneal shunt placement all patients had full neurological recovery with no complaints of headaches, lethargy, weakness, confusion or seizures. None of the cases had new subdural hematoma episodes after placement for a minimum of a two-year period (mean 26.1 months) (range 24.3-48.6 months). No postoperative complications were reported. The rates of postoperative hemorrhage, infection, distal catheter revision, and perioperative seizures was found to be zero percent. Shunt drainage was successful in all cases, draining 85% of the blood in the first 48 hours. There was no significant relationship between complications and the use of anticoagulants four weeks after surgery.

**Conclusions:**

Peritoneal shunts, though rarely used, are a viable option in the treatment of sub-acute/chronic subdural hematomas. When pursuing this treatment, this technique is recommended to mitigate the risks of repeat surgical intervention and lessen perioperative time in high-risk patients.

## Introduction

Recurrent/chronic subdural hematoma is one of the most frequent forms of intracranial hemorrhages treated by neurosurgeons, and one that may result in compression of brain parenchyma, mass effect, and subsequent neurological deficits and dysfunction. The prevalence of CSDH increases with age and is found most commonly afflicting the elderly population. Despite the frequency of this condition, research indicates that there is a lack of recommendations for standardized treatment [1]. While surgical management of the condition may be effective in some cases, complications associated with surgical techniques developed to manage the condition can result in significant risks to the patient’s health [1,4]. In the aging population, the existence of various comorbidities can affect the clinical outcome due to postoperative complications, including the increased risk for hemorrhage in patients receiving anticoagulant and antiplatelet therapy [2]. Because of the lack of guidance on the treatment of this condition, various authors have recommended a wide range of treatment options including: partial resection of the hematoma membrane, large craniectomy, extended craniotomy or excision of inner and outer parts of the hematoma membrane [4-5]. Overall, the options carry with them a host of risks for the patient and may not have a significant impact on improving patient outcomes and/or quality of life [5].

Although various recommendations for the treatment of chronic or recurrent subdural hematoma have been made, a review of the literature regarding treatment for this condition does indicate that craniotomy followed by drainage of the hematoma and replacement of the skull fragment is the most commonly utilized procedure for treating patients [4]. Decompressive craniectomy is also widely used to reduce intercranial pressure; however, in this procedure the skull fragment that is removed is not replaced [5]. Even though these interventions are among the most frequently employed, mortality rates for these procedures remain high at 60 to 80 percent [5]. Because of the high rate of mortality associated with craniectomy and craniotomy, recommendations for the use of preoperative trepanation and drainage are now being more extensively considered in practice [4]. Although, burr hole craniostomy is the most widely utilized technique, there is an approximate 10-20% recurrence rate requiring re-operation associated with this method [3]. The approach requires the placement of a burr hole in the skull to drain the hematoma and reduce pressure [6]. Because the efficacy of this approach has not been widely supported in the literature, it is primarily used to alleviate the pressure caused by a subdural hematoma in patients with comorbid conditions that preclude invasive surgery [6]. With the increasing incidence of chronic SDH in the aging population, there exists a need for new and refined treatment options that would provide both minimally invasive approaches while remaining clinically and fiscally efficient.

### Trepanation and peritoneal shunting

The use of trepanation (burr hole craniostomy) for the treatment of subdural hematoma does appear to provide some advantage in improving outcomes for patients with CSDH, especially those in which repeated invasive surgery is contraindicated. In one study, burr hole trepanation was compared with craniotomy in a group of 151 patients with subdural hematoma [7]. The results indicate that the success rate for the burr hole group was 64.3% compared with 52.3% for the craniotomy group [7]. Based on the data the authors conclude that trepanation may provide a more viable approach for addressing the needs of patients with subdural hematoma [7]. Further research on the topic suggests that trepanation followed by the placement of a peritoneal shunt may also provide additional support for improving patient outcomes [8]. Individual case reports regarding the use of peritoneal shunts for the treatment of subdural hematoma have been reported in the literature [1, 8]. Although peritoneal shunting is still in its infancy it may represent an important step forward in treating subdural hematoma.

Peritoneal shunting has been the treatment of choice for cerebrospinal fluid (CSF) diversion for decades [9]. Peritoneal shunts are commonly used in intercranial or spinal spaces to drain excess fluid in various sites and are often quite successfully utilized [8]. The peritoneal shunt is a relatively simple device that comprises three parts including the proximal tubing, the valve, and the distal tubing [8]. Although peritoneal shunts provide an effective tool for reducing the number of surgical procedures needed by the patient to reduce CSF pressure, these devices do require consistent monitoring and often fail within one year of placement [9]. Despite the fact that the literature regarding the use of peritoneal shunts for the treatment of subdural hematoma is scant, drainage of the hematoma typically occurs on a daily or weekly basis [8]. As a result, the placement of the peritoneal shunt has the potential to markedly reduce the number of surgical procedures needed to reduce intercranial pressure and may further be useful for patients with chronic or recurrent subdural hematoma.

### Purpose of the current research

As noted in the current literature the surgical treatment options for subdural hematoma carry with them considerable risk for the patient [1-2, 4-5, 6, 8]. Burr hole trepanation and peritoneal shunt placement may provide viable alternatives for reducing mortality associated with subdural hematoma while providing a cost-effective means for managing patients with recurrent hematomas [7-8]. While it is reasonable to argue that randomized clinical trials for evaluating the effectiveness of peritoneal shunt placement for subdural hematomas may raise some pertinent ethical concerns, it is possible to review patient charts to assess and evaluate treatments provided. As noted in the literature, techniques such as trepanation and peritoneal shunt placement are commonly used in patients who are unable to undergo invasive surgical procedures [1, 7-8]. For this reason, accumulated chart data regarding the use of peritoneal shunts for the treatment of subdural hematoma should provide important insight into the viability of this approach for mainstream treatment of this condition. Thus, the purpose of the current research is to provide a retrospective chart review that evaluates the use of peritoneal shunt placement in patients with CSDH and the subsequent outcomes in these patients. Informed consent was obtained from the patients for this study.

## Materials and methods

The method selected for this investigation involved a retrospective cohort review of 125 charts. The charts assigned for review included those of patients diagnosed with sub-acute/chronic subdural hematoma. Of the charts reviewed 18 patients were found to have a history of recurrent sub-acute or chronic subdural hematoma. All patients had undergone initial surgical treatment of their condition followed by peritoneal shunt placement to help alleviate intracranial pressure. Factors including age, the size of the hematoma, number of previous events, body mass index (BMI), complications, survival rates, and clinical course were all evaluated in an effort to comprehensively assess patient outcomes from the use of peritoneal shunts. The use of a retrospective cohort analysis provides important insight into peritoneal shunt treatment without creating additional ethical challenges for examining the use of this technique, including those associated with an experimental investigation of the approach. Table 1 describes the patient demographics.

**Table 1:** Patient Demographics

Patient Demographics	
Males	12
Females	6
Total patients	18
Patients with Parkinson's disease	3
Patients with Alzheimer's disease	3
Patients with CVD	10
Mean weight	72.1±9.3
Mean height	173±9.5
BMI	27.4±3.2
Previous trauma 20%	20%
Previous surgery for SDH	100%
ASA category II 6 patients III 11 patients IV 1 patient	

## Results

**Figure 1:**
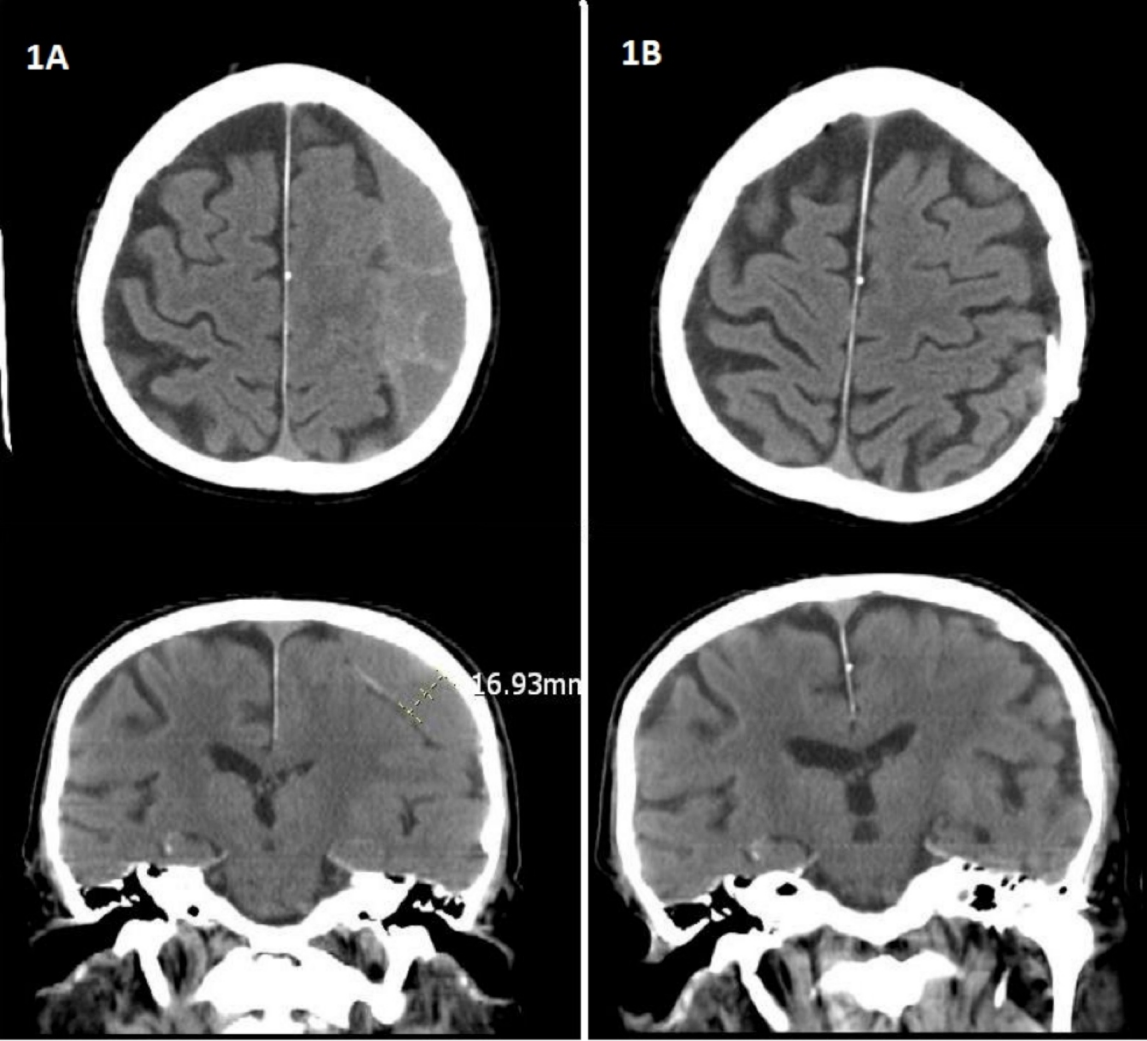
Brain CT Scan Images Figure 1A: Pre-op brain CT scan image demonstrating a large right subacute subdural hematoma causing subfalcine herniation, impending uncal herniation, right to the left shift of 1.0 cm mass effect and ipsilateral ventricles. Figure 1B: Brain CT scan demonstrating status post neurosurgical evacuation of right subdural hematoma. - Burr holes are present at the parietal convexity. Subdural drainage catheter, stable in position. Previously present right subdural air has resolved. No extra-axial fluid collections identified from the level of the foramen of Monro to the convexity. No acute intracranial hemorrhage.

The results of the chart analysis provide significant support for the use of peritoneal shunts for the treatment of subdural hematoma. In particular, the results indicate that all patients involved in the study experienced full neurological recovery following shunt placement. Additionally, the data demonstrates that patients with a peritoneal shunt in place did not experience a new subdural hematoma for a minimum of two years with the average period being 26.1 months and a range between 24.3 months and 48.6 months. Table 2 further details all evaluated secondary endpoints. The results further indicate that none of the patients experienced post-surgical complications and that shunt drainage was successful in all cases with 85 percent of the blood removed from the hematoma within 48 hours. Table 3 demonstrates complication rates. Four weeks following surgery there were no adverse complications associated with the use of anticoagulant medications. Figure 1 depicts preoperative and postoperative CT scans. Compared to Figure 1A (preoperative CT scan demonstrating large subacute subdural collection), Figure 1B demonstrates stable positioning of the drainage catheter with resolution of the hematoma and no signs of acute hemorrhage. 

**Table 2:** Summary of All Evaluated Secondary Endpoints

Summary of All Evaluated Secondary Endpoints	
Mean duration of surgery (minutes)	35.6 ± 12.5
Mean time until full mobility (days)	1.5 ± 1
Neurological recovery time	3.2 days ± 1.5 days
Correct intraperitoneal catheter position	100%
Incidence of recurring SDH at 2 years	30%

**Table 3:** Complication Rates

Complication Rates	
Hemorrhage	0%
Infection	0%
Perioperative seizure	0%
Distal catheter revision	0%
Proximal catheter revision	0%

## Discussion

Based on the data obtained from this retrospective chart review, peritoneal shunting is illuminated as a viable treatment of choice for patients with recurrent or chronic subdural hematoma. Current research indicates that the recurrence rate of subdural hematoma following surgery is between 9.2 and 26.5 percent [7]. Common surgical techniques used for the treatment of this condition have a mortality rate between 60 and 80 percent [6]. Given that the results of the current study indicate that none of the patients undergoing peritoneal shunt placement experienced morbidity or mortality as a result of the procedure, peritoneal shunt placement clearly has a marked advantage over existing surgical techniques. This surgical technique showed a comparatively safe and efficacious treatment strategy that is less invasive and cost-effective, for the management and treatment of chronic/sub-acute SDH and prevention of neurological decline secondary to recurrence of subdural hematoma.

### Limitations

Despite the fact that peritoneal shunt placement appears to be a superior choice for the treatment of chronic or recurrent subdural hematoma, it is important to note that the current research has some important limitations. In particular, the current research only identified 18 patients that had this procedure performed. This small sample size is not representative of the total population of patients who require treatment for subdural hematoma each year. As such, generalizing the findings for use in all patients with recurrent or chronic subdural hematoma is not advisable. Further, outcomes for the patients undergoing peritoneal shunt placement were not compared with outcomes for other patients undergoing procedures such as craniectomy and craniotomy. Information comparing outcomes may demonstrate that these surgical procedures provide some notable advantage over peritoneal shunt placement. With these issues in mind, it becomes evident that some type of comparison is required in order to fully understand the advantages of peritoneal shunt placement.

### Further research

Further research on the topic is clearly needed to elucidate the nuances of this treatment approach and to determine if it can and should be used in patients who are otherwise healthy enough to undergo craniectomy and craniotomy. In short, research is needed to compare peritoneal shunt placement with surgical techniques to determine if shunt placement is a viable option for patients who can undergo surgical treatment. Broadening the scope of retrospective chart reviews and comparing surgical groups with shunt placement groups may provide the most comprehensive data needed to determine if a randomized controlled trial can be ethically undertaken. By utilizing existing evidence to evaluate outcomes, it should be possible to draw more definitive conclusions from the research.

## Conclusions

Current surgical options for the treatment of subdural hematoma carry with them considerable complications, which can lead to increased mortality and morbidity. Peritoneal shunt placement appears to have some advantages for patients experiencing chronic or recurrent subdural hematomas. The current research provides promising support for the further investigation of peritoneal shunt placement for the treatment of subdural hematoma. While it is evident that additional research is needed to explore this technique, there is evidence to guide inquiry and the further development of methodologies that will help definitively demonstrate the efficacy of this technique.
